# Spectrophotometric Determination of Distigmine Bromide, Cyclopentolate HCl, Diaveridine HCl and Tetrahydrozoline HCl via Charge Transfer Complex Formation with TCNQ and TCNE Reagents

**Published:** 2015

**Authors:** Gehad Genidy Mohamed, Mahmoud Sabry Rizk, Eman Yousry Zaky Frag

**Affiliations:** *Chemistry Department, Faculty of Science, Cairo University, Giza, 12613, Egypt.*

**Keywords:** Spectrophotometry, Charge transfer, DDQ, Distigmine bromide, Cyclopentolate hydrochloride, Diaveridine hydrochloride, Tetrahydrozoline hydrochloride

## Abstract

The purpose of this investigation was directed to propose sensitive, accurate and reproducible methods of analysis that can be applied to determine distigmine bromide (DTB), cyclopentolate hydrochloride (CPHC), diaveridine hydrochloride (DVHC) and tetrahydrozoline hydrochloride (THHC) drugs in pure form and pharmaceutical preparations via charge-transfer complex formation with 7,7,8,8-tetracyanoquinodimethane (TCNQ) and tetracyanoethylene (TCNE) reagents. Spectrophotometric method involve the addition a known excess of TCNQ or TCNE reagents to DTB, CPHC, DVHC and THHC drugs in acetonitrile, followed by the measurement of the absorbance of the CT complexes at the selected wavelength. The reaction stoichiometry is found to be 1:1 [drug]: [TCNQ or TCNE]. The absorbance is found to increase linearly with concentration of the drugs under investigation which is corroborated by the correlation coefficients of 0.9954-0.9981. The system obeys Beer’s law for 6-400, 20-500, 1-180 and 60-560 µg mL^-1^ and 80-600, 10-300, 1-60 and 80-640 µg mL^-1^ for DTB, CPHC, DVHC and THHC drugs using TCNQ and TCNE reagents, respectively. The apparent molar absorptivity, sandell sensitivity, the limits of detection and quantification are also reported for the spectrophotometric method. Intra- and inter-day precision and accuracy of the method were evaluated as per ICH guidelines. The method was successfully applied to the assay of DTB, CPHC, DVHC and THHC drugs in formulations and the results were compared with those of a reference method by applying Student’s t and F-tests. No interference was observed from common pharmaceutical excipients.

## Introduction

Analytical methods that were used for the quantitative determination of drugs played a significant role in the evaluation and interpretation of bioavailability, bioequivalence, pharmacokinetic and fully validated analytical methods to yield reliable results that could be satisfactory interpreted. Analytical methods and techniques were constantly being changed and improved, in many instances; these methods were at cutting edge of the technology. Also, it was important to emphasize that each analytical technique had its own characteristics, which will vary from drug to drug. 

**Figure 1:**
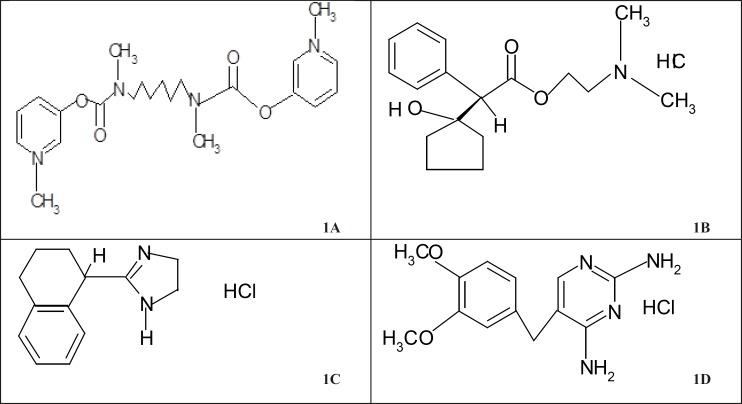
Structure of (1a) DTB, (b) CPHC, (1c) THHC and (1d) DVHC drugs

Distigmine bromide (DTB, Figure 1a) was designated by chemical abstracts as 3,3-[hexamethylenebis(methyliminocarbonyloxy)]bis(1-methylpyridinium) dibromide.

It was identified according to the previously reported method (1). Demecarium bromide was quantitatively determined by colourimetric method based on the reaction with potassium cobaltothiocyanate. The blue colour was extracted with 1,2-dichloroethane and the absorbance was measured at 320 and 625 nm. The method was also applied to other structurally related cholinesterase inhibitors such as pyridostigmine bromide and distigmine bromide (2).

A screening procedure was developed for the identification and quantification of distigmine bromide in serum samples by using liquid chromatography (LC)–electrospray ionization (ESI)-mass spectrometry (MS). An assay could be performed with simple solid phase extraction using a cation exchange cartridge column by carrying out selected ion monitoring analysis in the positive ion detection mode. The method was used for the detection, quantification, and confirmation of distigmine bromide in serum (3)_._

Cyclopentolate hydrochloride (CPHC, Figure 1b) was nomenclated according to the IUPAC system as: benzene acetic acid, α-(1-hydroxycyclopentyl)-2-(dimethylamino) ethyl ester hydrochloride, (±)- or 2-(dimethylamino)ethyl(±)-1-hydroxy-α-phenylcyclopentaneacetate hydrochloride. The structure was given in Figure (1b).

Cyclopentolate hydrochloride is an anticholinergic drug that blocks the response of the sphincter muscle of the iris and the accommodative muscle of the ciliary body to stimulation by acetylcholine (4). According to chromatographic technology application note, cyclopentolate enantiomers were separated by HPLC using a CHIRAL-AGP column and CHIRAL-AGP guard column, a mobile phase of 4% propan-1-ol in 10 mM – sodium phosphate buffer of pH 7 and detection at 225 nm (5). ?^-1^) and f.i.d. hexadecanol was used as internal standard, and the compounds were silyated with trifluorobis(trimethylsilyl)acetamide–chlorotrimethylsilane (99:1) before injection (6)**.**

 One visible spectrophotometric and another fluorimetric method have been developed for the determination of CPHC from bulk and ophthalmic solutions. Spectrophotometric method was based on the formation of greenish blue coloured species on treatment with Folin-Ciocalteu (FC) reagent in alkaline medium, while fluorimetric method was based on the hydrolysate solution of CPHC in 0.5 sodium hydroxide on excitation at 366 nm emits yellowish blue fluorescence measured at 475 nm (7). Another method had been developed for the determination of CPHC in pure form and in its pharmaceutical preparations based on its reaction with ammonium metavanadate reagent in acidic medium for the required reaction time at boiling water bath and after the reaction was completed, the unconsumed reagent was back determined by titration against ferrous ammonium sulphate using N-phenylanthranilic acid indicator (8). Four ion - exchangers (Fx-Rt (I), Fx-TPB (II), Cp3-PMA (III) and Cp3-PTA (IV)) of antispasmodic and anticholinergic drugs, flavoxate hydrochloride (FxCl), 2-piperidinoethyl-3-methyl-4-oxo-2-phenyl-4h-1-benzo-pyran-8-carboxylate hydrochloride, CPHC and (2-(dimethylamino)ethyl(RS)–(1-hydroxycyclo-pentyl)phenylacetate) hydrochloride were synthesized and incorporated into poly(vinylchloride)-based membrane electrodes for the quantification of FxCl and CPHC in different pharmaceutical preparations. The influence of membrane composition on the potentiometric response of the membrane electrodes was found to substantially improve the performance characteristics. The practical utility of the sensors was demonstrated by the determination of FxCl and CPHC in pure solutions and pharmaceutical preparations using standard additions and potentiometric titration (9).

 Tetrahydrozoline hydrochloride (THHC) has the structure given in Figure 1c. It has the IUPAC name 2-[(1RS)-1, 2, 3, 4-tetrahydronaphthalen-1-yl]-4,5-dihydro-1H-imidazole hydrochloride (10). HPLC method was developed for the simultaneous determination of ofloxacin (OFX), tetrahydrozoline hydrochloride (THHC), and prednisolone acetate (PAC) in ophthalmic suspension using propylparaben (POP) as the internal standard. The detection was carried out using UV–visible detector at 210 nm for OFX and THHC and 254 nm for POP (internal standard) and PAC (11). Samples, containing THHC (I), were diluted with mobile phase if required and portions were analysed on a 5 microm LiChrospher Si 60 column with aqueous 70% methanol, containing 0.03% triethylamine and 0.02% acetic acid as mobile phase (1 mL min^-1^) and detection at 254 nm. Calibration graphs were linear from 1-20 and 12.5-500 μg mL^-1^ of I, with intra– and inter–day RSD on samples of 1.3 – 11.8%. Average recoveries of 20-50 µg mL^-1^ of I was added to ophthalmic solutions were 98.9-99.9% (12).

For UV spectrophotometric analysis, the eye drop formulations were diluted with mobile phase to bring the concentrations of the two ingredients within the calibration ranges and second – order derivative spectra were recorded from 210 to 300 nm. The THHC (I) and the fluorometholone (II) were measured at 226 and 282 nm, respectively. Calibration graphs were linear from 5-20 and 20-60 µg mL^-1^, respectively, for I and II. For HPLC analysis, the eye drop formulation was treated with lidocaine (internal standard) and diluted to 25 mL with mobile phase. Portions (20 µL) were analyzed on a 3 µm Partisil 5 ODS column, with H_2_O/acetonitrile/methanol (1:5:5) as mobile phase (1.5 mL min^-1^) and detection at 220 nm. Calibration graphs were linear from 3-20 and 10-60 µg mL^-1^, respectively, for I and II. The results on two formulations by both methods were 99.2-101.6% of the labelled values (13?^-1^) consisted of aq.40 % methanol containing 20 mM-Na octane-1-sulphonate and 10 mM-NN-dimethyloctylamine. Calibration graphs were rectilinear and the coefficient of variation (n = 7) and the average recovery was 1.64 and 101.1%, respectively (14). The sample was diluted with the mobile phase for analysis on a column of Hypersil C8 and the detection was done at 222 nm. 4-dimethylaminobenzaldehdye was used as the internal standard. Excipients and potential degradation products did not interfere (15). Tetrahydrozoline (I) and its possible decomposition product, N-ethylamino-1,2,3,4-tetrahydro-1-naphthamide (II), were seperated by HPLC on a column of micro bondapak C18 with Na_2_B_4_O_7_-KH_2_PO_4_ buffer solution of pH 7.0 – acetonitrile (3:2) as mobile phase and detection at 254 nm. The detection limit was 300 µg of I injected, with rectilinear response in the range from 0.5 to 10 µg (16).

Diaveridine hydrochloride (DVHC, Figure 1d) was designated by chemical abstracts as: 5-[(3,4-dimethoxyphenyl)-methyl]-2,4-pyrimidinediamine hydrochloride; 2,4-diamino-5-veratryl-pyrimidine hydrochloride; 2,4-diamino-5-(3,4-dimethoxy-benzyl)pyrimidine hydrochloride (17). Its structure was given in Figure (1d).

Charge transfer complexation has been a well-known interaction since the early discoveries made by Benesi and Hildebrand (18), through the pioneering work of Briegleb (19) and the valence-bond, and molecular orbital descriptions put forth by Mulliken (20), and Dewar and Lepley (21), respectively. The molecular interactions between electron-donating pharmaceutical compounds and electron acceptors are generally associated with the formation of intensely colored charge-transfer complexes, which absorb radiation in the visible region (22). A variety of electron donating compounds have been reported to yield charge-transfer complexes with various acceptors. The rapid formation of these complexes leads to their utility in the development of simple and convenient spectrophotometric methods for these compounds (23–30).

In the present paper, the development of simple and precise spectrophotometric method for determination DTB, CPHC, DVHC and THHC drugs via charge transfer complex formation with some p -acceptors namely TCNQ and TCNE as chromogenic reagents was reported. Different experimental factors affecting these reactions are optimized and then Beer’s law is carried out. This spectrophotometric method was applied for the determination of the drugs under investigation in pure form and their dosage forms. Confirmation of the applicability of the proposed spectrophotometric method was validated according to the International Conference on Harmonization (ICH) guidelines for the determination of the four drugs in pure form and in tablet dosage forms. No interference was observed from the tablet additives.

## Experimental

*Materials*

All chemicals and reagents were of analytical reagent grade and some of them were used as such without any further purification. They included distigmine bromide that provided by Arab Drug Company, Egypt. Reagents used included 7,7,8,8-tetracyanoquinodimethane (TCNQ) and tetracyanoethylene (TCNE) which were supplied from Aldrich. Glacial acetic acid was supplied from Merck. Absolute ethanol was supplied from Adwic, while *n*-propanol and acetonitrile (AR) were supplied from Aldrich. Chloroform, methanol, acetone, tetrahydrofuran, 1,4–dioxane, n-butanol, methylene chloride and dimethyl formamide were supplied from El-Nasr Company. The distigmine bromide pharmaceutical preparations were bought from Ubreted tablets, 5 mg/tablet, (Arab Drug Company, Egypt) 

The cyclopentolate hydrochloride pharmaceutical preparations were purchased from Colircuci Ciclopejico Eye drops, 10 mg mL^-1^ (Alcon cusi _⁄ _S.A). The tetrahydrazoline hydrochloride pharmaceutical preparations were purchased from Visine Eye drops 0.05% (Pfizer Egypt S.A.E.).

*Solutions*

5x10^-3^ and 7.8x10^-3^ mol L^-1^ solutions of DTB, CPHC, DVHC and THHC were prepared in ethanol on using TCNQ and TCNE reagents, respectively. Solutions were always freshly prepared by dissolving the accurate weighed amount in the proper amount of ethanol. 0.1%(w/v) Solution of TCNQ and TCNE reagents was prepared by dissolving the accurate weighed amount of 100 mg TCNQ or TCNE in 100 mL acetonitrile. All solutions must be protected from light by keeping them in a dark coloured quickfit bottles during the whole work. The water was always twice distilled from all glass equipments. Redistillation was carried out from alkaline permanganate solution.

*Equipments*

The spectrophotometric measurements were carried out using the manual spectronic 601 (Melton Roy Company), and Perkin Elmar automated spectrophotometer in the wavelength range from 200 to 900 nm.

*Procedures*

 Parameters affecting spectrophotometer determination of DTB, CPHC, DVHC and THHC via charge transfer complex reaction with TCNQ and TCNE reagents:

*Selection of the suitable wavelength*

 In calibrated 5 mL volumetric flask, different aliquots, containing 0.1-0.5 mL of 0.1 %(w/v) of DTB, CPHC, DVHC and THHC drugs were added to 0.1-0.5 mL of 0.1%(w/v) TCNQ or TCNE solutions. The volumes were completed to the mark with acetonitrile. The absorption spectra of the resulted CT complexes were scanned in the wavelength range λ = 350-900 nm from which the best wavelength for each drug was selected.

*Effect of time and temperature*

 To select the optimum time and temperature for the complex formation, 0.1–0.5 mL of 0.1% (w/v) of DTB, CPHC, DVHC and THHC drugs were added to 0.1-0.5 mL of 0.1%(w/v) of TCNQ or TCNE solutions. The volumes were completed to the mark with the applicable solvent. First the absorbance was measured at different time intervals in the range of 0–120 minutes. Second the absorbance was measured at different temperatures in the range from 0 to 60 °C.

*Effect of TCNQ or TCNE concentration*

 0.1-0.5 mL of 0.1% (w/v) of working solutions of DTB, CPHC, DVHC and THHC drugs were added to different volumes of 0.1% (w/v) of TCNQ or TCNE solutions. The volumes of TCNQ and TCNE reagents were ranged from 0.1-2 mL and completed with acetonitrile to 5 mL. The absorbance was measured at the specific wavelength for each drug.

*Effect of organic solvents*

 The same above procedure for drugs was followed using different organic solvents. Ethanol, chloroform, n-propanol, methanol, 1,4–dioxane, petroleum ether, 1,2-dichloroethane, acetonitrile and dimethyl formamide were tried to decide which of them causes more colour development.

*Stoichiometric ratio of the CT- complexes formed*

*The continuous variation method:*

A series of solutions were prepared by adding different volumes of 0.1% (w/v) of TCNQ or TCNE to 5x10^-3^ or 7.8x10^-3^ mol L^-1^ of pharmaceutical drugs, respectively, so that the total number of moles is kept constant. The procedures were followed as above and the absorbance data obtained were plotted against mole fraction of each drug.

*The molar ratio method*

 1 mL of (5x10^-3 ^or 7.8x10^-3^ mol L^-1^) of pharmaceutical drugs were added to different volumes of 0.1% (w/v) of TCNQ or TCNE reagents ranged from 0.1 to 3 mL in 5 mL volumetric flask and the absorbance was measured against ratio of reactants.

*Validity of Beer's law*

 Suitable volumes of 0.1% (w/v) TCNQ or TCNE were added to different concentrations of 0.1% (w/v) of drugs (0.1-2.5 mg mL^-1^) and ( 0.01-2.8 mg mL^-1^) in case of TCNQ and TCNE reagents, respectively. The mixtures were completed up to 5 mL with acetonitrile. The absorbance of the coloured complex products were measured at the specific wavelengths against reagents blank prepared similarly without drugs.

*Between– day measurements*

 In order to prove the validity and the applicability of the proposed method and the reproducibility of the results obtained, four replicate experiments at different concentrations of pharmaceutical drugs were carried out. Using the above mentioned procedures, the absorbance of the two samples were measured daily for four days and the results were recorded to make statistical calculations. 

*Application on some pharmaceutical preparations*

10 Tablets were accurately weighed and the average tablet weight was calculated. The tablets were then ground to a fine powder. An equivalent weight was dissolved in the least amount of ethanol. The resulting solutions were shaken, filtered through a Whatmann No. 1 filter paper and washed with ethanol. The filtrate and washings of drugs were collected in 100 mL measuring flask.

 Different concentrations of pharmaceutical drugs were added to suitable volumes of (0.1% w/v) TCNQ or TCNE reagents. The volumes were made up to the mark with acetonitrile in 5 mL calibrated measuring flask. The absorbance was measured at λ_max_ = 842 or 415 nm for pharmaceutical drugs using TCNQ or TCNE reagents, respectively, against acetonitrile reagent blank. 

## Results and Discussion

π

**Figure 2:**
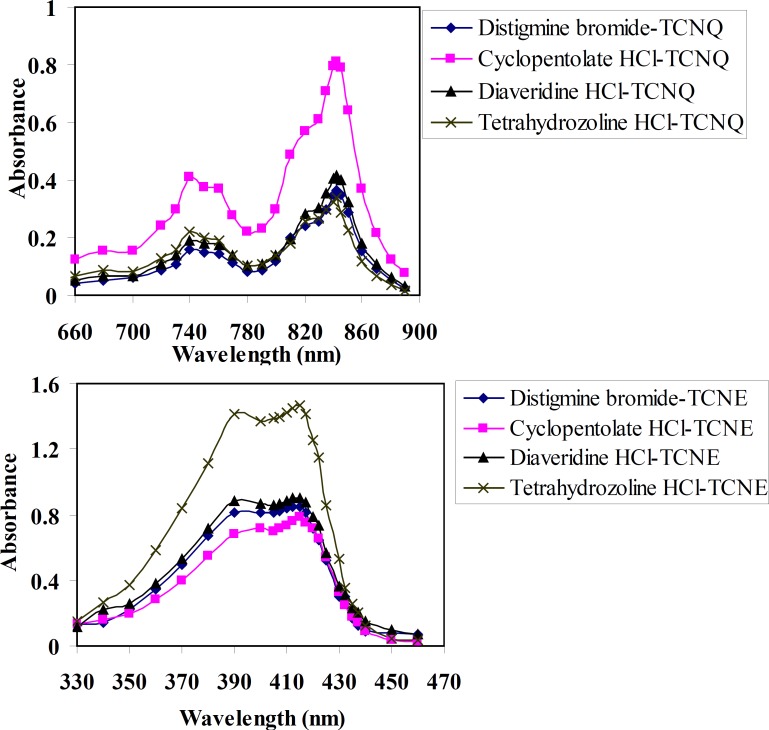
Absorption spectra of charge-transfer complexes of distigmine bromide, cyclopentolate HCl, diaveridine HCl and tetrahydrozoline HCl with (a) TCNQ and (b) TCNE reagents in acetonitrile.

Figure (2a) shows the absorption spectra of TCNQ reagent with DTB, CPHC, DVHC and THHC CT complexes in acetonitrile solvent. A solution of drugs and TCNQ reagent in acetonitrile solvent yield an intense greenish colour which has a characteristic long-wavelength absorption bands, frequently with two maxima at λ = 745 and 842 nm in the electronic spectrum. The absorption spectra of TCNE reagent with DTB, CPHC, DVHC and THHC in acetonitrile result in the formation of an intense yellow product which exhibits an absorption maximum at λ = 415 nm as given in (Figure 2b).

The predominate chromogen with TCNQ or TCNE reagents is the green or yellow radical anion A* which was probably formed by the dissociation of an original donor-acceptor (DA) complex with DTB, CPHC, DVHC and THHC drugs.

**Figure:**
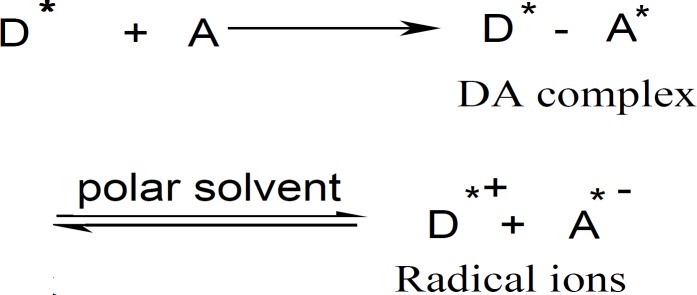


Where D = donor and A = acceptor and A* = TCNQ* or TCNE* in polar solvent. The dissociation of the DA complex is promoted by the high ionizing power of the acetonitrile solvent (31-33).

*Effect of solvent*

In order to select the suitable solvent for CT complex formation, the reaction of TCNQ and TCNE reagents with DTB, CPHC, DVHC and THHC drugs is made in different solvents. These solvents included acetonitrile, chloroform, n-propanol, methanol, 1,4–dioxane, 1,2-dichloroethane, petroleum ether, ethanol and dimethylformamide. It is found that acetonitrile is considered to be an ideal solvent for the colour reaction as it offers solvent capacity for TCNQ and TCNE and gives the highest yield of the radical anion as indicated by high ε values. This is because it possesses the high dielectric constant of all solvents examined; a property which is known to promote the dissociation of the original CT complex to radical ions *i.e*. the dissociation of donor–acceptor complex is promoted by the high ionizing power of the solvent.

*Effect of reagents concentration*

**Figure 3:**
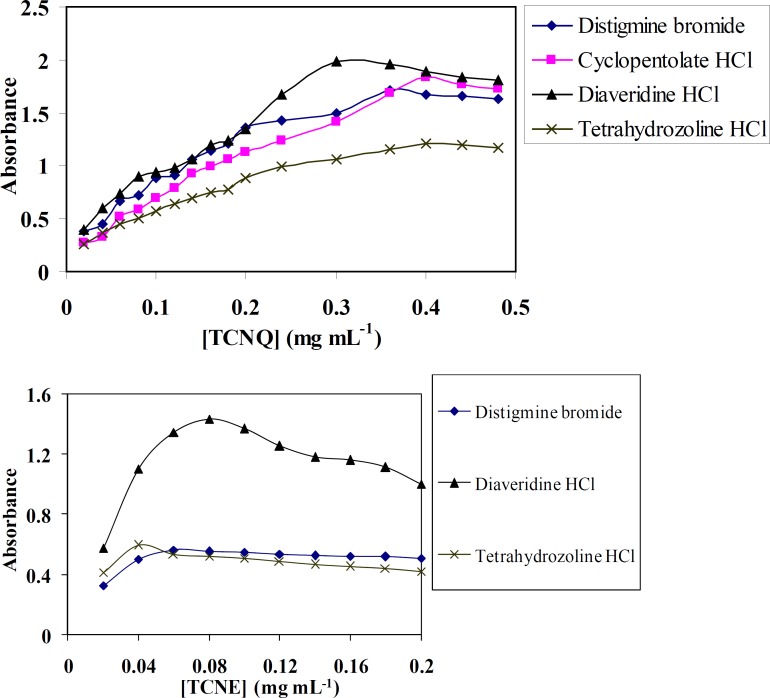
Effect of (a) TCNQ and (b) TCNE concentrations on the formation of distigmine bromide, diaveridine HCl and tetrahydrozoline HCl-CT complexes in acetonitrile

Figure (3) shows the effect of 0.1% (w/v) TCNQ and TCNE reagents on the quantitativeness of their reactions with DTB, CPHC, DVHC and THHC drugs. It is found that, when various concentrations of TCNQ or TCNE solutions added to a constant concentration of DTB, CPHC, DVHC and THHC drugs, it is obvious that (0.3-0.4) or (0.04-0.08) mg mL^-1^ of TCNQ or TCNE solutions, respectively, is found to be sufficient for quantitative determination of the drugs under study.

*Effect of time*

**Figure 4:**
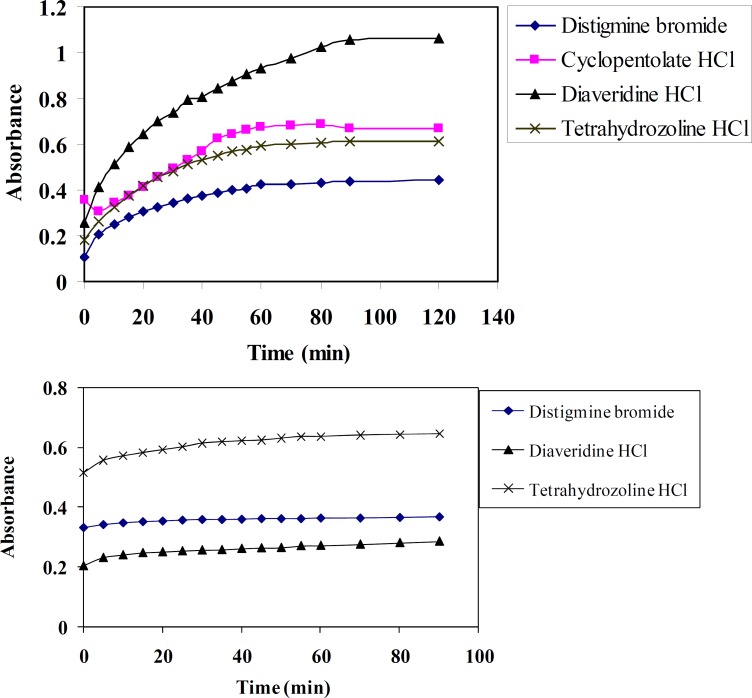
Effect of time on the absorbance of CT complexes of distigmine bromide, diaveridine HCl and tetrahydrozoline HCl with (a) TCNQ and (b) TCNE reagents in acetonitrile

Time of reaction has a pronounced effect on quantitativeness of the reaction between DTB, CPHC, DVHC and THHC (electron donor) and TCNQ or TCNE reagents (electron acceptors). The optimum reaction time is determined spectrophotometrically at different time intervals and at λ_max _= 842 or 415 nm for TCNQ and TCNE reagents, respectively. Figure (4) shows that complete colour development is attained after 60 and 30 minutes for TCNQ and TCNE reagents, respectively. Also the colour remains stable for one day at least using these reagents.

*Effect of temperature*

**Figure 5:**
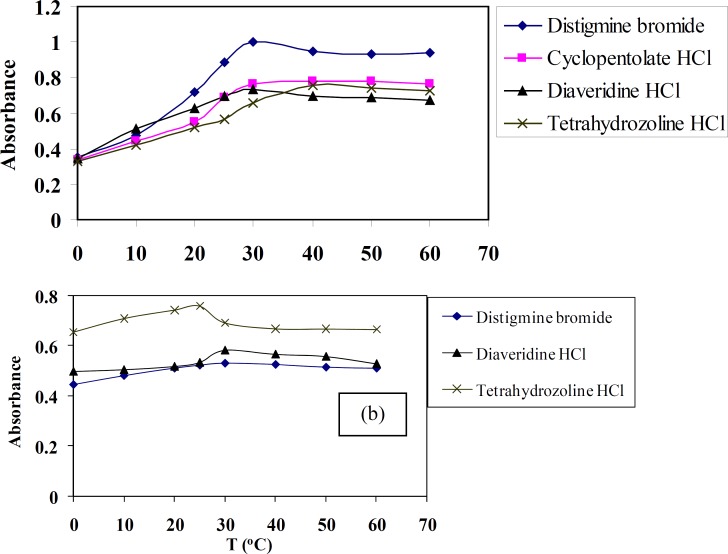
Effect of temperature (0-60 °C) on the absorbance of CT complexes of distigmine bromide, diaveridine HCl and tetrahydrozoline HCl with (a) TCNQ (t = 30 min, λ = 842 nm) (b) TCNE (t = 30 min, λ = 415 nm) reagents in acetonitrile

The aim of studying this factor using spectrophotometric method is to check the effect of temperature on the quantitativeness of these reactions. The effect of temperature in the range of 0 to 60 ºC on TCNQ or TCNE reactions with DTB, CPHC, DVHC and THHC drugs was studied. The absorbance of these CT complexes is measured at 842 and 415 nm for TCNQ and TCNE reagents, respectively, against the blank solution prepared without the drug. The effect of temperature on these CT complexes is shown in Figure 5. The given results show that the absorbance attains a maximum colour at temperature 30-40 or 25-30 ºC for TCNQ and TCNE reagents, respectively. The colours of the reaction products CT complexes are remained constant for at least 24 hour.

*Stoichiometry of the CT complexes*

**Figure 6:**
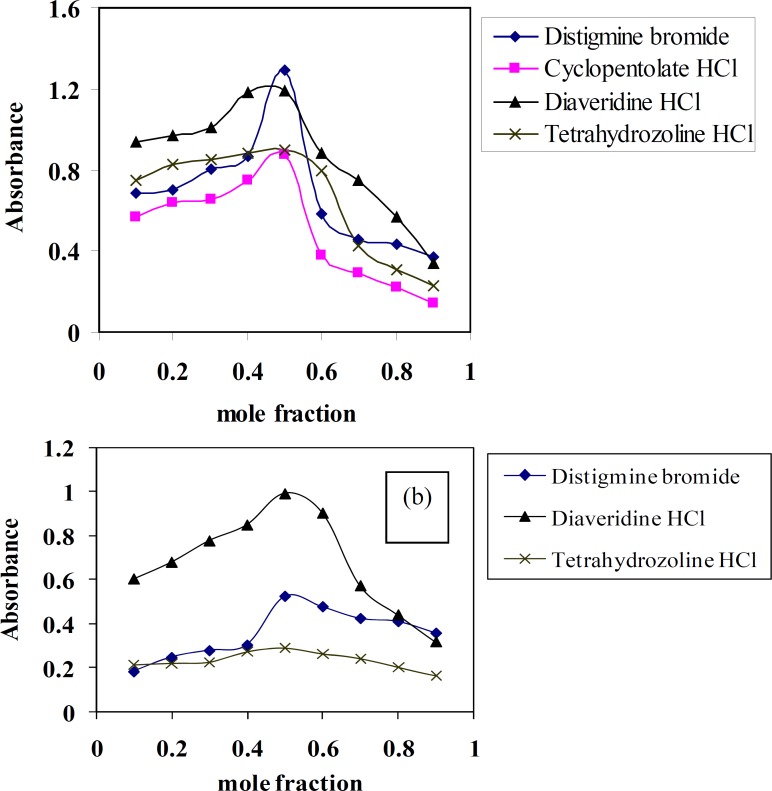
Job's method for distigmine bromide, diaveridine HCl and tetrahydrozoline HCl CT complexes with (a) TCNQ and (b) TCNE reagentsin acetonitrile

**Figure 7:**
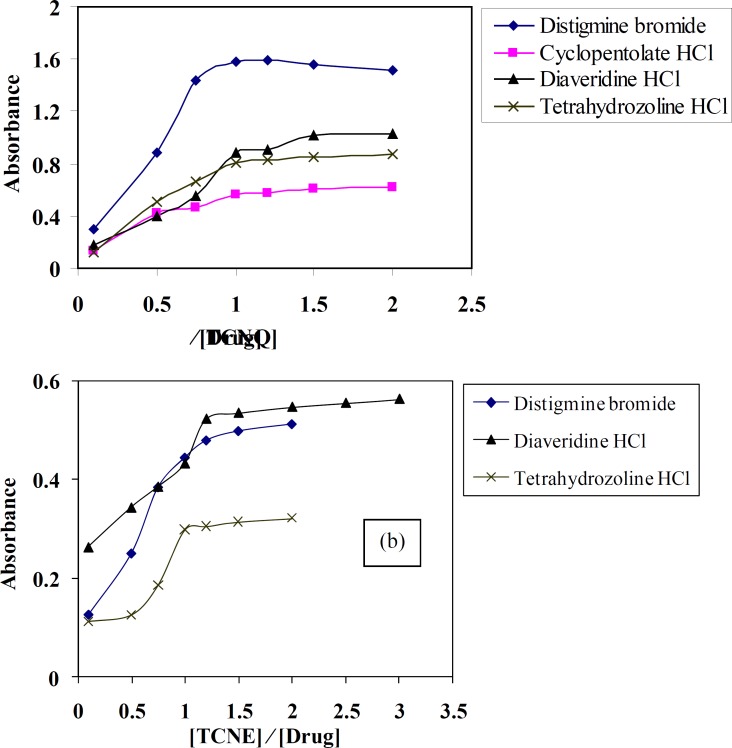
Molar ratio of distigmine bromide, diaveridine HCl and tetrahydrozoline HCl-CT complexes with (a) TCNQ and (b) TCNE reagents in acetonitrile

**Figure 8:**
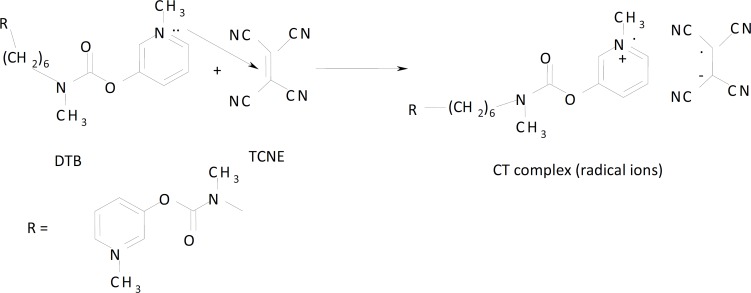
CT complex formation between DTB drug with TCNE

**Figure 9:**
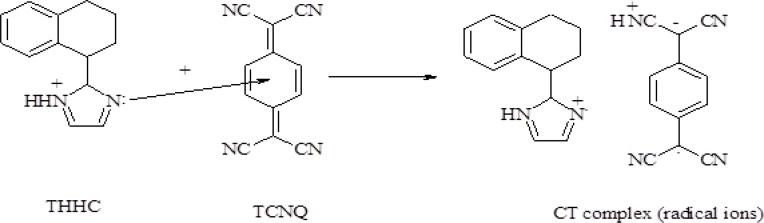
CT complex formation of THHC with TCNQ

Molar ratio and Job’s continuous variation methods (34, 35) are applied in order to determine the suitable ratio between DTB, CPHC, DVHC and THHC drugs and TCNQ or TCNE reagents. Figures (6, 7) show that the interaction between these drugs and reagents occurs in equimolar basis, *i.e* the two straight lines are intersected at 1:1 [Drug]: [Reagents]. This means 1:1 CT complexes were formed between the drugs and TCNQ or TCNE reagents. The CT complexes formed between TCNQ or TCNE reagents and DTB, CPHC, DVHC and THHC drugs takes place through the migration of H^+^ ion to one of the four cyano groups in TCNQ and TCNE reagents to form positive ion which associate with the phenolate anion to form ion pairs (31-33π∗^31^-^33^, ^36^). Figures (8, 9) represent the proposed CT complexes of DTB with TCNE and THHC drug with TCNQ as examples (36). 

*Obeyence to Beer’s law*

 After the selection of suitable pH, solvents, reagent concentrations, reaction time, temperature, and ratio it is also important to know the concentration limits of DTB, CPHC, DVHC and THHC drugs at which these reactions are quantitative. Consequently, it is easy to apply this spectrophotometric method to determine these drugs under investigation quantitatively in pharmaceutical formulations via its reaction with electron acceptor reagents like TCNQ or TCNE reagents.

Table (1) shows the results of studying quantitativeness of the reaction between DTB, CPHC, DVHC and THHC drugs with TCNQ and TCNE reagents under selected optimum conditions. It is found that, Beer’s law is valid over the concentration ranges from 6 to 400 and 80 to 600 μg mL^-1 ^of DTB drug using TCNQ and TCNE reagents, respectively. While, the calibration curve is linear in the concentration range of 20-500 μg mL^-1^ of CPHC drug using TCNQ reagent.

While the calibration curves are linear in the concentration range of 1-180 and 1-60 μg mL^-1 ^of DVHC drug using TCNQ and TCNE reagents, respectively. In addition, the calibration curves are linear in the concentration range of 60-560 and 80-640 μg mL^-1^ of THHC drug using TCNQ and TCNE reagents, respectively. Table 1 shows the slope, intercept, correlation coefficient, Sandell sensitivities, molar absorptivity (ε), range of error, standard deviation and relative standard deviation. The small values of Sandell sensitivity indicate the high sensitivity of the proposed method in the determination of the drugs under investigation. Four replicate measurements are performed at different concentrations of DTB, CPHC, DVHC and THHC drugs. The relative standard deviation and the range of error values are calculated and found that the small values of them indicate the high accuracy and high precision of the proposed spectrophotometric method.

*Between-day determination of TB, CPHC, DVHC and THHC drugs.*

In order to prove the validity and applicability of the proposed method and reproducibility of the results obtained, four replicate experiments at four concentrations of DTB, CPHC, DVHC and THHC drugs were carried out. Tables (2, 3) show the values of the between-day relative standard deviations for different concentration of the drugs, obtained from experiments carried out over a period of four days. It is found that, the between day relative standard deviations are less than 1%, which indicates that the proposed method is highly reproducible and TCNQ and TCNE reagents are successfully applied to determine DTB, CPHC, DVHC and THHC drugs via the charge transfer reaction.

*Spectrophotometric determination of DTB, CPHC, DVHC and THHC drugs in different pharmaceutical preparations*

The spectrophotometric determination of DTB, CPHC, DVHC and THHC drugs via their reaction with TCNQ and TCNE (strong electron acceptors) reagents are carried out. The results obtained are given in Tables (4, 5). These data show that, the determined concentration of DTB, CPHC, DVHC and THHC drugs by the proposed method are closed to that calculated from the applied standard method. In order to check the confidence and correlation between the suggested spectrophotometric procedures and the official method (1, 4, 10) for determination of DTB, CPHC, DVHC and THHC drugs, it is better to do the F- and t-tests for all the results (Tables 4, 5). 

The calculated F- and t-tests at the 95% confidence level do not exceed the theoretical values indicating non significant difference between the proposed and official method. The small values of SD and RSD indicate the reliability, accuracy and precision of the suggested procedures.

*Validation of the proposed method*

*Linearity, detection, and quantitation limits*

Following the proposed experimental conditions, the relationship between the absorbance and concentration was quite linear in the concentration ranges given in Table 1. The intercept (a), slope (b), correlation coefficient (r), molar absorptivities (ε), and Sandell sensitivity values are summarized in Table 1. The percentage recoveries of the pure drugs using the proposed methods compared with that given by the reported methods are illustrated in Tables (3, 4). The validity of the proposed spectrophotometric method was evaluated by statistical analysis between the results achieved from the proposed method and that of the reported methods (1, 4, 10). Regarding the calculated t-test and F-test (Tables 3, 4), it is concluded that there is no significant difference between the proposed and reported methods regarding accuracy and precision. The detection limit (LOD) is defined as the minimum level at which the analyte can be reliably detected for the four drugs was calculated using the following equation (37, 38), and listed in Table 1:

LOD = 3s / k

Where s is the standard deviation of replicate determination values under the same conditions as for the sample analysis in the absence of the analyte and k is the sensitivity, namely the slope of the calibration graph. The limits of quantization, LOQ, is defined as the lowest concentration that can be measured with acceptable accuracy and precision (37, 38), and the data are listed in Table 1.

LOQ = 10 s / k

*Accuracy and precision*

The accuracy and precision of the proposed spectrophotometric method were evaluated by carrying out four replicate analyses on pure DTG, CPHC, DVHC and THHC drug solutions at four different concentration levels within the working range. Percentage relative standard deviation (RSD%) as precision and standard deviation (SD) as accuracy of the proposed spectrophotometric method were calculated (Tables 2, 3). The relative standard deviation values were found to be less than 2% in all cases, indicating good repeatability of the suggested method. The intra- and inter-day precision and accuracy results show that the proposed method has good repeatability and reproducibility (Tables 2, 3).

*Ruggedness and robustness*

The ruggedness of the proposed method was assessed by applying the procedures using two different instruments in the different laboratories. It is found that the RSD did not exceed 2.0% which indicate that laboratory-to-laboratory variation was found to be reproducible. Robustness of the procedures was assessed by evaluating the influence of small variation of experimental variables, *i.e*., concentrations of TCNQ and TCNE reagents and reaction time, on the analytical performance of the method. In these experiments, one experimental parameter was changed while the other parameters were kept unchanged, and the recovery percentage was calculated each time. The small variations in any of the variables did not significantly affect the results. This provided an indication of the reliability of the proposed method during routine work. 

*Interference studies*

This study was performed in order to show the effect of possible interfering species on the reaction of the four drugs under investigation with the TCNQ and TCNE reagents. The selectivity of the proposed spectrophotometric method was investigated by observing any interference encountered from some common excipients of the pharmaceutical formulations such as starch and sugars like lactose, sucrose, glucose and maltose. It was observed that these excipients did not interfere with the proposed method. So, the proposed spectrophotometric method is able to determine the four drugs under investigation in the presence of common excipients. 

## Conclusion

The present study described the development and validation of spectrophotometric assay for the determination of DTG, CPHC, DVHC and THHC drugs based on their CT reaction with TCNQ and TCNE reagents. In this assay, the CT reaction was carried out in acetonitrile. The assay described herein offered the advantages of reduction in the consumption of organic solvents in the CT-based spectrophotometric analysis, accordingly reduction in the exposures of the analysts to the toxic effects of organic solvents and reduction in the analysis cost. Although the proposed assay was developed and validated for DTG, CPHC, DVHC and THHC drugs, however, it is also anticipated that the same methodology could be used for essentially any analyte that can exhibit CT reaction. 
